# Positive Effects of a Resveratrol-Based Nutraceutical in Association with Surgical Scleroembolization: A Pilot Retrospective Clinical Trial

**DOI:** 10.3390/jcm13102925

**Published:** 2024-05-15

**Authors:** Emilio Italiano, Giada Ceccarelli, Giovanna Italiano, Fulvio Piazza, Rossana Giulietta Iannitti, Tiziana Puglisi

**Affiliations:** 1 Centro Genesi, Via Libertà, 90144 Palermo, Italy; eitaliano@gmail.com; 2 S&R Farmaceutici S.p.A., 06083 Bastia Umbra, Italy; g.ceccarelli@srfarmaceutici.com; 3 Policlinico Brescia, Unità Operativa di Urologia, 25128 Brescia, Italy; doc.ita93@gmail.com; 4 Azienda Ospedaliera “Cervello-Villa Sofia“, 90146 Palermo, Italy; piazzafulvio@gmail.com (F.P.); dott.tpuglisi@gmail.com (T.P.)

**Keywords:** varicocele, scleroembolization, Resveratrol, infertility

## Abstract

**Background:** Varicocele still today represents a common cause of infertility in young men. The treatment strategy remains a surgical approach such as scleroembolization; however, the complete restoration of spermatic parameters afterward requires an average of six or more months to fully regain optimal seminal parameters. Recently, many studies have demonstrated the beneficial effects of Resveratrol in male fertility, given its potential anti-inflammatory, antiapoptotic, and mitochondrial effects. Therefore, Resveratrol-based nutraceuticals could be promising as an adjuvant to mitigate subfertility in patients with varicocele. **Methods:** In the present study, we retrospectively analyzed the effects of the administration of a Resveratrol-based nutraceutical after the scleroembolization procedure. The improvement of sperm quality in terms of number, motility, and morphology were considered to be the study’s main endpoints. A spreadsheet program was used for data analysis, and a *p*-value of <0.05 was considered significant. **Results:** We found a statistically significant improvement in the spermatic parameters (sperm count and total motility) and an increase in normal sperm after only 4 months of treatment. The supplementation with a Resveratrol-based nutraceutical associated with the surgical procedure showed encouraging results if compared to data from a control group and the results reported in the literature linked to scleroembolization practice alone. In fact, there was a clear improvement in the seminal parameters at 4 months. **Conclusions:** This suggests the positive impact of the Resveratrol-based nutraceutical in synergizing with scleroembolization in reducing the time needed to fully recover sperm function.

## 1. Introduction

Varicocele represents the most common correctable cause of infertility in young men [1]. A varicocele is clinically defined as an abnormal dilation and enlargement of the scrotal venous pampiniform plexus. The incidence of varicocele is approximately 15–20% when considering the entire male population but increases to 40% when considering infertile males [2], indicating a strong connection between clinically significant varicocele and male infertility. Indeed, although not usually accompanied by painful symptoms, varicoceles are clinically significant, as they are the most commonly identified cause of abnormal semen analysis, including low sperm count, reduced sperm motility, and increased abnormal sperm morphology. However, the specific mechanism by which varicocele affects sperm production, structure, and function remains uncertain, despite several theories.

As depicted in the EAU Guidelines for “Sexual and Reproductive Health” [3], the treatment of choice remains a surgical strategy for patients with clinical varicocele and altered semen parameters. To date, many surgical methods are available for the treatment of varicocele, such as scleroembolization and microsurgical subinguinal varicocelectomy. No clear data indicate the best surgical treatment, but we know that it could improve the symptoms and sperm parameters, offering recurrence rates of less than 4%. It is well known that varicocele repair significantly improves both natural and in vitro fertilization procedures and live birth and pregnancy rates, as well as sperm count, total and progressive motility, morphology, and DNA fragmentation rates. In general, after varicocelectomy, up to 70% of patients have improved sperm quality, with an average of a minimum of two spermatogenic cycles [4] and spontaneous pregnancy occurring between 6 and 12 months [5]. However, the degree of restoration of the seminal parameters and fertility after varicocelectomy is known to vary according to the ages of patients. Specifically, adolescents between 15 and 19 years of age have a complete recovery of the ability to conceive, with a 100% pregnancy rate within the first year of marriage [6], while improvement in the seminal parameters may include only 50% of adults (>19 years of age) undergoing varicocelectomy [7]. 

Several studies in the literature have previously described the role of inflammation in the pathogenesis of varicocele by describing the state of testicular and sperm inflammation in animal models, particularly in mice, as well as in men with varicocele, with or without alterations in the seminal parameters [5]. In this context, according to the varicocele pathophysiology, a group of cytosolic receptors called nucleotide oligomerization domain (NOD)-like receptor family pyrin domain containing 3 (NLRP3) inflammasomes may be also involved in this mechanism [8]. It is worth noting that, from a mechanistic point of view, oxidative stress and testicular apoptosis are also believed to play significant roles [9]. 

Recently, both clinical [10,11,12] and preclinical [13,14,15] studies have demonstrated that botanical derivatives like Resveratrol (trans-3,5,4′-trihydroxystilbene), a naturally occurring polyphenolic molecule found in several plants, such as *Polygonum cuspidatum* and the seeds of grapes, peanuts, blueberries, bilberries, and cranberries, can have a positive effect on sperm quality and can be useful in numerous chronic pathologies. Indeed, several in vivo and in vitro studies have demonstrated that this compound has anti-inflammatory, antioxidant [16,17], and antiapoptotic properties [18].

From a mechanistic point of view, these actions depend on different pathways; among them, Resveratrol is involved in the activation of antioxidant enzymes, such as catalase and superoxide dismutase, having a crucial role in the lipid damage of human spermatozoa [19]. Furthermore, Resveratrol shows an anti-inflammatory activity by the inhibition of COX2 and NF-KB [20] and protects cells from DNA damage and apoptosis by modulating anti- and proapoptotic mediators, leading to an increase in sperm vitality, sperm DNA integrity, and an improvement in oxidative stress [21].

Moreover, Resveratrol can improve mitochondrial activity and trigger a series of molecular mediators able to affect cellular metabolic mechanisms [22]. Resveratrol also plays a role in calcium signaling cascades and in modulating the mitochondrial intrinsic apoptotic pathway.

This in vitro evidence is corroborated by in vivo preclinical studies on rat models, highlighting the promising use of Resveratrol as a nutraceutical substance adjuvant to mitigate subfertility in varicocele [8,21,23]. As an example, Mendes et al. demonstrated that Resveratrol partially reduces testicular apoptosis and reverses alterations in the sperm motility and promotes mitochondrial activity and DNA integrity [23], as well as improves the oxidative status and sperm vitality [21]. According to the obtained results, Hajipour et al. [8] suggested that Resveratrol might be an adjuvant therapeutic option in patients with varicocele by virtue of decreasing inflammatory events and apoptosis in varicocele-induced rats.

**Figure 1:**
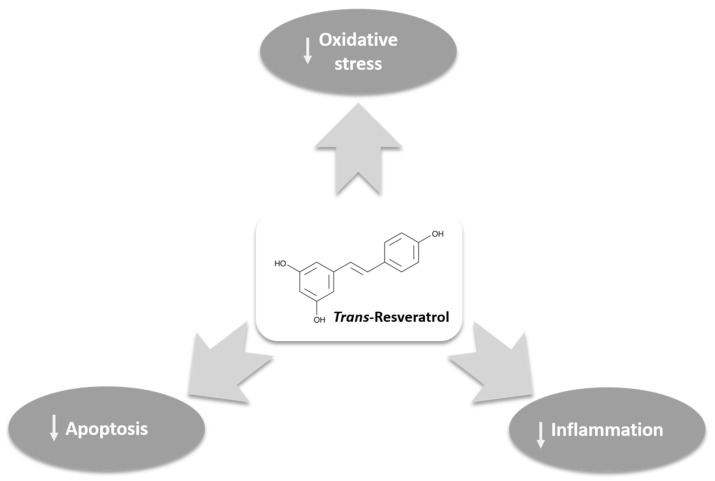
Mechanism of action of Resveratrol in the management of varicocele.

In recent years, only preliminary human clinical research has indicated that nutritional interventions based on Resveratrol can significantly counteract male infertility [10]. All these data suggest the possibility of improving varicocele clinical manifestations and recovery of the seminal parameters by reducing oxidative, inflammatory, and proapoptotic stimuli. Moreover, it corroborates the potential use of Resveratrol in managing impaired male fertility and varicocele (Figure 1) and suggests the need for extensive studies in this regard. 

In this context, in this retrospective study, the effects of a nutraceutical containing Resveratrol and magnesium dihydroxide (trademark Revifast^®^), vitamin B12, vitamin B6, vitamin D, and folic acid were evaluated on the spermatic function after surgical scleroembolization. 

The purpose of this study is to investigate whether Resveratrol-based nutraceutical therapy in association with varicocele repair can improve the clinical practice in terms of timing and extent of recovery of the seminal parameters and what impact it may have on fertility.

## 2. Materials and Methods

This retrospective, controlled study was conducted at the Reproduction Center “Genesi” in association with “Villa Sofia-Cervello” Hospital of Palermo between 2021 and 2023. Patients were admitted to our institution with a diagnosis of bilateral or left-side varicocele and treated with transcatheter percutaneous scleroembolization or through the basilic vein or the right femoral vein. 

As the normal clinical practice, antibiotic therapy with 4–5 days of I generation cephalosporin was suggested at discharge. Patients eligible for analysis in the study group were chosen from those who received daily supplementation with 150 mg of Resveratrol and magnesium dihydroxide (trademark Revifast^®^, Prolabin&Tefarm, Perugia, Italy), 2.5 µg of vitamin B12, 1.4 mg of vitamin B6, 25 µg of vitamin D, and 400 µg of folic acid (two tabs of nutraceutical/day for 4 months), while control patients were chosen between those who did not receive any nutraceutical supplementation. 

Inclusion criteria: According to the registry of men submitted to scleroembolization, men with grade II or grade III bilateral and left-side varicocele on physical evaluation after standing for 5 min [24] were considered potentially eligible for the retrospective study, which was restricted to patients seeking consultation at the included center. 

Exclusion criteria: According to clinical guidelines, men with known causes for male subfertility other than varicocele, such as cryptorchidism, treated cancer, surgery of the scrotum and of the genital tract, or hypogonadotropic hypogonadism, were excluded from varicocele repair. Azoospermic men were also excluded from the study. 

This retrospective analysis was restricted to patients preliminary submitted in our institution to CDU to confirm a bilateral or left-side varicocele by the occurrence of a continuous left SVR before undergoing a retrograde or anterograde phlebography of the internal spermatic vein and scleroembolization. Clinical examination of the genitals, CDU, and laboratory tests were performed at baseline and at 4 months after the surgical procedure. In total, data from 86 patients (mean age 30.12 ± 7.21) were collected for the study group and 20 patients (mean age 26.49 ± 5.92) for the control group.

Variables and outcomes: Patients’ data at baseline were complete with an anthropometric assessment, including weight, height, and body mass index (BMI). As the usual clinical practice, blood samples were collected in the early morning for determination of the follicle-stimulating hormone (FSH), luteinizing hormone (LH), testosterone (T), and prolactin. Semen analyses were performed in our institutions according to the WHO criteria (2010) [25], and ejaculates were collected through masturbation in both centers by a certified seminologist after 3–5 days of sexual abstinence. All participants considered for the statistical analysis (N = 56) had at least two semen analyses at baseline and at 4 months, provided that no fever, no genital tract infection, and no major trauma or surgical procedures occurred between the two examinations. As the normal clinical practice, following the EAU Guidelines [3], if the semen analysis was abnormal according to the WHO criteria, a second analysis was performed (reporting the result as the mean ± SD). The semen parameters included the total spermatozoa (10^6^), total sperm motility (%), forward sperm motility (%), non-forward sperm motility (%), and normal sperm morphology (%).

The primary study outcome was the improvement of sperm quality in terms of number, motility, and morphology. The secondary outcomes obtained were pregnancy (%) and the resolution of SVR after repair.

Scrotal ultrasonography: All patients underwent an evaluation with EchoColorDoppler both before the intervention and after 3–4 months during the follow-up.

Varicocele repair: The surgical procedure we used to treat varicocele was transcatheter percutaneous scleroembolization, a radiologic technique [26], in which the access employed is by the right common femoral vein after local anesthesia or through the basilic vein.

After a limited fluoroscopy to minimize radiation exposure to patients, the spermatic venous incompetence at its origin from the renal vein was confirmed. The catheter was inserted in the spermatic vein and the tip placed between the distal internal spermatic vein and the pampiniform plexus. The reflux in the pampiniform plexus was verified by fluoroscopy during the Valsalva maneuver, with a wide visualization of the pampiniform plexus. After protection of the testis by hand pressure on the left pampiniform plexus, we used from 1 to 3 vials of lauromacrogol 400 (Atossiclerol 3%).

Post-procedure care: As the usual clinical practice, patients were observed for 1 h after the procedure before discharge. They were advised to rest for 72 h, avoiding Valsalva pressure. After 30 days, the success of the procedure was determined by clinical examination and CDU recording of a retrograde flow. We deemed that the scleroembolization procedure was effective if the complete absence of reflux in the spermatic vein was recorded by CDU in the standing position after varicocelectomy. CDU was repeated after 3 months to confirm the results.

Statistical analysis: A spreadsheet program was used for data analysis. All continuous variables were examined for normality with the D’Agostino–Pearson test. Normally distributed variables were expressed as the mean ± standard deviation (SD). The *t*-test or the Mann–Whitney test was used for comparison as appropriate. Qualitative variables were expressed as the number and proportions and were compared to the chi-square test or Fisher’s exact test as appropriate. All the collected variables and outcomes were compared between the baseline and T_4_ in a bivariate analysis. 

## 3. Results

Retrospective data were collected from 86 patients relative to the period between 2021 and 2023 in two Sicilian andrological or reproductive centers. The demographic and clinical characteristics of the included patients are shown in Table 1.

**Figure 2:**
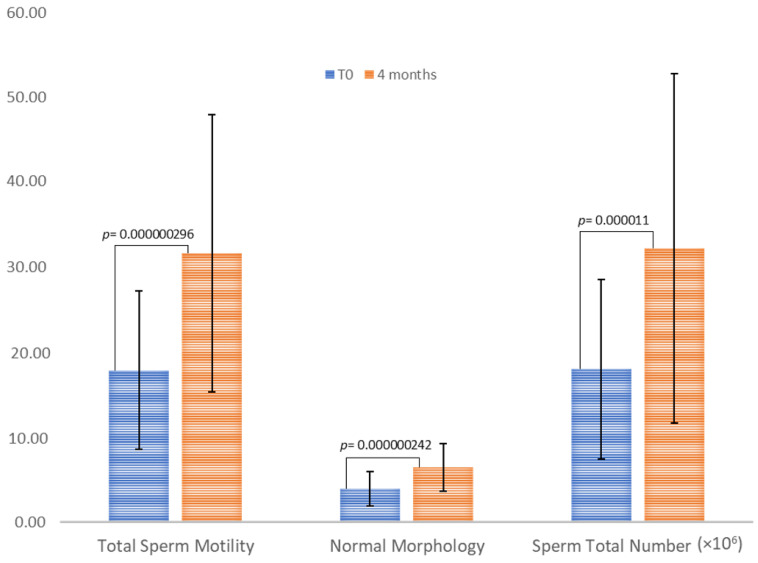
Improvements of the semen parameters at 4 months. Data are reported as the mean ± SD.

The laboratory assessment after 4 months of post-surgical treatment with a nutraceutical containing Resveratrol and magnesium dihydroxide (trademark Revifast^®^), vitamin B12, vitamin B6, vitamin D, and folic acid (two tabs/day for 4 months) showed a statistically significant improvement in the total sperm count (32.30 ± 20.60 × 10^6^/ejaculate vs. baseline 18.03 ± 10.59 × 10^6^/ejaculate, 91% average increase), total motility (17.91 ± 9.29 × 10^6^/ejaculate vs. 31.75 ± 16.34 × 10^6^/ejaculate, 75% average increase), and an increase in normal morphology (*p* = 0.000000242, 64.6% average increase) (Table 2 and Figure 2). These results can be compared to retrospective data collected from 20 control patients with Dubin varicocele II or III who did not receive post-surgical nutraceutical treatment (baseline demographic data in Supplementary Table S1). Table 3 shows a nonsignificant increase in the total sperm count (mean increase 12%, *p* = 0.43), total sperm motility (mean increase of 15%, *p* = 0.43), and normal morphology (mean increase of 15%, *p* = 0.39) after 4 months post-scleroembolization. To be noted, these mean increases are markedly smaller than the significant increases observed in the study group treated with the Resveratrol-based nutraceutical (Table 2). In the study group, a significant % resolution of varicocele detected by Doppler post-scleroembolization (95%) was observed, as well as an overall spermiogram improvement (82%) and spontaneous pregnancies (16%) at 6 months after scleroembolization (Table 4). The pregnancy rate at 6 months was 16%, considering the complete study group, but in only 17 patients was this desired, leading to a 70% pregnancy rate among the desired pregnancy group (Table 4). Compared to the control patients, the Resveratrol treatment led to a significant number of patients in which the overall spermiogram improved (82% study group vs. 50% control group, *p* = 0.00166) (Table 4).

Furthermore, comparing these retrospective results with other trials evaluating the impact of scleroembolization practice alone at 6 months [7,27], the Resveratrol-based nutraceutical treatment intervention associated with surgical scleroembolization analyzed in this retrospective study clearly shows an interesting improvement in terms of sperm quality and recovery time of normal sperm parameters in just 4 months, so as to justify further investigation of this association to clinical practice (Table 5).

## 4. Discussion

The impact of varicocele on sperm production and fertility is well known, and many mechanisms have been proposed to be involved in the pathophysiology. Inflammation, apoptosis, and oxidative stress all impact the seminal parameters and are all altered by varicocele. Many recent studies have shown that Resveratrol can impact the seminal parameters and affect fertility overall. 

Indeed, in subfertile men, varicocele is associated with oxidative sperm damage and sperm DNA fragmentation, which are hypothesized to contribute to subfertility [28]. Treatment with Resveratrol is associated with decreased oxidative stress, inflammation, and sperm DNA fragmentation [19,20] and thus increases the likelihood of pregnancy and could be useful in improving the clinical parameters even in patients with more difficult recovery from surgery.

A recent pilot clinical study showed a significant increase in the number, concentration, and motility of spermatozoa among idiopathic infertile men [10]. Other studies have also shown biological benefits of Resveratrol in infertile women undergoing ICSI-IVF procedures [29], possibly by promoting mitochondrial biogenesis in granulosa cells [30]. 

Although the exact mechanism of Resveratrol remains to be understood in the spermatogenesis process, the possible anti-inflammatory and antiapoptotic effects could surely impact the seminal parameters. Noteworthy are the mitochondrial effects promoted by Resveratrol. It has been clearly demonstrated that the mitochondrial membrane potential and function is strictly related to sperm motility [28], and there is also evidence from other studies that Resveratrol affects the molecular mechanisms by which calcium signaling impacts the functional outcomes in granulosa cell metabolism and mitochondrial biogenesis and that these mechanisms may impact follicle maturation [30]. Therefore, we can assume that Resveratrol may also similarly impact Sertoli cells, which contribute to normal spermatogenesis, mainly due to their influence on the nutrient supply, maintenance of cell junctions, and assist in gametic cell mitosis and meiosis. 

However, only further preclinical studies will be able to highlight the complexities of sperm differentiation and how Sertoli cell modulation by Resveratrol may contribute to promoting healthy spermatogenesis and possibly also correct mitochondrial dysfunction and a decline in sperm motility during the spermatogenesis process.

All these considerations are interesting, because mitochondria also control many crucial functions of spermatozoa, providing the energy needed for their motility and ensuring a minimum concentration of reactive oxygen species, which, in the physiological range, contribute to sperm maturation, capacitation, and acrosome reaction. On the other hand, functional or structural dysfunction at the level of sperm mitochondria generates an overproduction of reactive oxygen with consequent oxidative stress and impaired energy production, leading to sperm DNA damage, decreased sperm motility, and worsening of the semen parameters and thus reduced male fertility. These aspects underline the pivotal role of mitochondria in male fertility [31].

The Resveratrol-based nutraceutical studied in this clinical trial also contained magnesium, vitamin D, vitamin B6, vitamin B12, and folic acid, nutraceutical substances that may have an impact on the semen parameters. 

As an example, the literature has reported the positive effects of vitamin B12 on sperm quality in terms of improved sperm number and sperm motility and reduced sperm DNA damage [32]. Folic acid is also reported to significantly increase sperm density in patients with oligospermia or asthenospermia [33]. On the contrary, so far, no statistical correlations have been found between the amount of seminal plasma vitamin B6 and the seminal parameters [34]. Moreover, vitamin D is also directly implicated in ameliorating the semen quality and sperm motility, male reproductive potential, and testosterone levels [35,36]. Probably, this is due to the ability of vitamin D to promote the synthesis of ATP through the cAMP/PKA pathway [36]. However, to date, the role of vitamin D and other vitamins in male fertility still remains debated [34,37]. 

This present pilot retrospective trial shows an overall statistically significant spermiogram improvement in just 4 months and an increase in the spontaneous pregnancy rate after scleroembolization, clearly demonstrating the added benefit of a nutraceutical approach based on Resveratrol and vitamins in clinical practice. 

The combined treatment led to a significant average increase in the total sperm number, total motility, and morphology that was not significant in the control patients. However, a strict comparison between the study group and control group could not be evaluated, considering that not all the demographic baseline data were homogeneous (Supplementary Table S1). It is interesting to note that, if you consider the sperm total number, starting from comparable baseline values (18.03 ± 10.59 in the study group vs. 16.45 ± 8.38 in the control group, *p* = 0.63), only a significant increase was found for the study group (T_0_: 18.03 ± 10.59, T_4_: 32.30 ± 20.60, *p* = 0.000011 vs. T_0_: 16.45 ± 8.38, T_4_: 19.50 ± 9.81 in the control group, *p* = 0.43) (Table 2 and Table 3).

It is interesting to note that this combined approach allows restoring the semen parameters in a shorter time with respect to the average time reported as the normal practice (generally, more than 6 months). Furthermore, in support of this, one can consider the outcomes obtained in studies evaluating the impact of scleroembolization practice alone [7,27]. It is important to point out that the seminal parameter values derived from the work of Mancini [7] and D’Andrea [27] and used for the comparison were taken at 6 months from the groups with positive outcomes in scleroembolization practice (disappearance of left spermatic vein reflux (SVR)), which would therefore reasonably have had better restoration of the seminal parameters than the groups with a persistent SVR at the scrotal color-Doppler ultrasound (CDU) after varicocele repair. Therefore, these results clearly show the interesting improvement of the seminal parameters at 4 months rather than those reported in the literature at 6 months [27] and, in some cases, even to a greater extent (Table 5). In particular, the average increase in the sperm parameters at 4 months from the present work was interestingly better with respect to the average increase in the sperm parameters in the works of Mancini [7] and D’Andrea [27] at 6 months in terms of the sperm total motility (75% increase vs. 6.8% increase; see Table 5) and normal morphology (64.6% increase vs. 42.9% and 59.6% increases; see Table 5). On the contrary, there is not a better result in terms of the average increase (%) of the sperm total number (91% increase vs. 479.2% and 122.3% increases; Table 5). 

Moreover, in support of this, the results reported by the EAU Guidelines 2024 [3] regarding the surgical resolution of varicocele depicted significant improvements in terms of the pregnancy rates and total sperm count found between T0 and T12 [38] and also significative differences in sperm motility and normal sperm morphology between scleroembolized and untreated patients [39] at 6 or 12 months, showing how time is important for a complete recovery. 

Considering these obtained results, this study may suggest that nutraceutical therapy based on Resveratrol and vitamins in combination with varicocele repair may have a positive effect on the sperm quality and recovery time of normal sperm parameters, with a beneficial effect on the couple’s fertility. 

This study, however, does have some limitations. The study is a pilot retrospective study, meaning it lacks blinding and randomization. A comparison with a small control group of 20 patients with Dubin II and III varicocele who did not receive any post-surgical nutraceutical treatment was evaluated; however, it was not possible to effectively compare all the variables, because not all baseline data were homogeneous between the two groups (Supplemental Table S1). Changes in the semen parameters were evaluated at 4 months after varicocele repair, while a second observation time might have been of interest in better evaluating the recovery of the parameters. Furthermore, a follow-up at 6 months after varicocele repair to obtain the pregnancy rate might be too short to properly assess the outcome and could be extended in a future work. Further prospective placebo-controlled studies could confirm the role of Resveratrol-based nutraceuticals in improving clinical practice in terms of timing and the extent of recovery of the seminal parameters in men with clinical varicocele submitted to varicocelectomy and can assess the true extent of this synergistic approach.

## 5. Conclusions

A Resveratrol-based nutraceutical therapy combined with surgical scleroembolization may lead to improved seminal parameters and may reduce the time needed to fully recover sperm function. The exact mechanisms are still poorly understood; perhaps, the improvement of oxygenation is due to surgical repair together with the impact of Resveratrol on cellular metabolism and could create a positive effect in restoring the physiological process of spermatogenesis. Altogether, these effects may contribute to reduce the time needed to fully recuperate sperm function, benefiting the fertility potential of couples. 

## Figures and Tables

**Table 1:** Demographic data of the study patients at baseline.

Parameters	Study Group
Age	30.12 ± 7.21
Height (cm)	174.72 ± 4.27
Weight (Kg)	79.69 ± 7.77
BMI (Kg/m^2^)	26.09 ± 2.24
Smoking (%)	37.5% (yes) 62.5% (no)
Varicocele Dubin (1–3) (%)	54.65% (Degree 2) 45.35% (Degree 3)
Anatomic variants (%)	13.33% (yes) 86.67% (no)
Bilateral varicocele (%)	4.44%
Left-side varicocele (%)	94.44%
Right-side varicocele (%)	1.11%
Intraoperative adverse events (iAEs) (%) *	1.11%
Coil (%)	5.56%
Glue (%)	37.97%
LH	4.01 ± 1.40
FSH	4.46 ± 2.24
Prolactin	12.34 ± 16.63
Testosterone (T)	5.22 ± 3.48
Sperm Total Number (×10^6^)	18.03 ± 10.59
Sperm Total Motility	17.91 ± 9.29
Forward Motility	12.93 ± 13.71
Non-Forward Motility	6.84 ± 7.31
Immobility	79.12 ± 16.42
Normal Morphology	3.92 ± 2.04
Leucocytes < 1 mld (%)	7.78%

Data are reported as *n* (%) or mean ± SD, as appropriate. * iAE: swelling of the inguinal canal, pain, and of embolism.

**Table 2:** Outcome comparison of the study group at baseline and 4 months.

Outcome	Baseline Study Group (*n* = 56)	4 months Study Group (*n* = 56)	Average Increase %	*p*
Sperm Total Number (×10^6^)	18.03 ± 10.59	32.30 ± 20.60	91%	0.000011
Sperm Total Motility	17.91 ± 9.29	31.75 ± 16.34	75%	0.000000296
Normal Morphology	3.92 ± 2.04	6.45 ± 2.80	64.6%	0.000000242

Data are reported as the average increase (%) or mean ± SD, as appropriate.

**Table 3:** Outcome comparison of the control group at baseline and 4 months.

Outcome	Baseline Control Group (*n* = 20)	4 months Control Group (*n* = 20)	Average Increase %	*p*
Sperm Total Number (×10^6^)	16.45 ± 8.38	19.50 ± 9.81	12%	0.43
Sperm Total Motility	38.00 ± 17.85	42.37 ± 16.40	15%	0.43
Normal Morphology	61.69 ± 31.73	70.79 ± 23.90	15%	0.39

Data are reported as the average increase (%) or mean ± SD, as appropriate.

**Table 4:** Improvements (%) and outcomes after scleroembolization and treatment with the nutraceutical.

Outcome	(*n* = 86)	(*n* = 20)	*p*
Resolution of varicocele detected by Doppler post-scleroembolization *n* (%) ^1^	82 (95%)	18 (90%)	0.6926
Improvement of spermiogram *n* (%) ^1^	70 (82%)	10 (50%)	0.00166
Spontaneous pregnancy *n* (%) ^2^	14 (16% on total number) (70%; desired *n* = 17)	1 (5% on total) (20%; desired *n* = 5)	0.13230

Data are reported as *n* (%). ^1^ Outcome evaluated at 4 months. ^2^ Outcome evaluated at 6 months.

**Table 5:** Improvements (%) after scleroembolization at 4 and 6 months.

Outcome	%Incr (4 Months) Italiano et al. 2024	%Incr (6 Months) D’Andrea et al. 2019 [27]	%Incr (6 Months) Mancini et al. 2019 [7]	Δ (%)
Sperm Total Number	91%	479.2%	122.3%	−388.2 [27] −31.3 [7]
Sperm Total Motility	75%	-	6.8%	+68.2 [7]
Normal Morphology	64.6%	42.9%	59.6%	+21.7 [27] +5 [7]

Data are reported as the average increase (%) of the sperm total count, sperm total motility, and normal morphology. Patients’ demographic data [7,27]: Age (years) = 35.0 (30.8–39); left varicocele grade II or III Dubin at physical examination and a continuous left-side SVR > 3 cm/s at scrotal CDUS, submitted to VR by retrograde internal spermatic vein embolization. D’Andrea et al. 2019 [27]. Age (years) = 16.6 (13–19); left varicocele grade II or III Dubin with Tanner stage 3–5. Mancini et al. 2019 [7].

## Data Availability

Data supporting the reported results are available upon request. To request the data, contact the corresponding author of the article.

## Supplementary Materials

The following supporting information can be downloaded at https://www.mdpi.com/article/10.3390/jcm13102925/s1: Table S1: Control patient’s demographic data at baseline.
