# Supplementary material for: Positive Effects of a Resveratrol-Based Nutraceutical in Association with Surgical Scleroembolization: A Pilot Retrospective Clinical Trial
Source: J Clin Med. 2024 May 15;13(10):2925. doi: 10.3390/jcm13102925 (PMC11122415; doi:10.3390/jcm13102925)
Supplement: Supplementary file 1 [file jcm-13-02925-s001.zip › jcm-2961095-supplementary.pdf]

Supplementary File

Table S1. Control patient's demographic data at baseline

Parameters	Control Group
Age	26.49 ± 5,92
Height (cm)	176.51 ± 7.08
Weight (Kg)	75.40 ± 8,84
BMI (Kg/m^2)	24.18 ± 2,37
Smoking (%)	27%(yes)<br>73% (no)
Varicocele Dubin (1-3) (%)	22.22% (Degree 2)<br>77.68% (Degree 3)
Anatomic variants (%)	35.29% (yes)<br>64.71% (no)
Bilateral varicocele (%)	3%
Left-side varicocele (%)	97%
Right-side varicocele (%)	0%
Intraoperative adverse events (iAEs) (%)	3%
Coil (%)	13%
Glue (%)	90%
Sperm Total Number (×10^6)	16.45 ± 8.38
Sperm Total Motility	38.00 ± 17.85
Normal Morphology	61.69 ± 31.73
Leucocytes < 1 mld (%)	6%

Data are reported as n (%) or mean ± SD, as appropriate. *iAE: swelling of inguinal canal, pain and of embolism.
